# Supplementary material for: Pathophysiological Significance of Neutrophilic Transfer RNA-Derived Small RNAs in Asymptomatic Moyamoya Disease
Source: Cells. 2021 May 1;10(5):1086. doi: 10.3390/cells10051086 (PMC8147334; doi:10.3390/cells10051086)

2D Structure	Local AU	Position

```
110                                      138
                       8mer-1a
 5'-ttgagctgggaggagtcttCAATGTGGGa-3' utr
                      |  | |||||||
 3'-uccuuaggauuggcgaucuGGUACACCCu-5' tRF
                 16151413      7 6 5 4 3 2
              3' Pairing        Seed
```

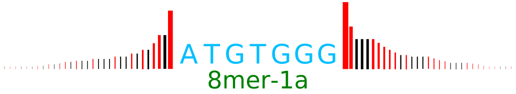

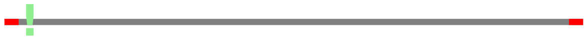

2D Structure	Local AU	Position

```
20                                       48
                        8mer-1a
 5'-tgGAATTTCTGTGCCTCT--TTTATGTGGGa-3' utr
      |||  |:||:  :||  ||    ::|||||||
 3'-ucCUU-AGGAU-UGGCGAUCUGGUACACCCu-5' tRF
                 16151413      7 6 5 4 3 2
              3' Pairing        Seed
```

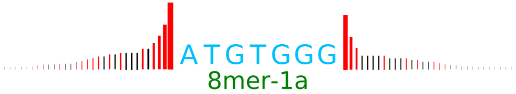

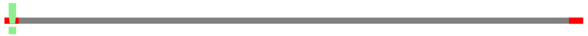

Supplement: Supplementary file 1 [file cells-10-01086-s001.zip › cells-1149161 3rd supplementary materials/Data Supplements/Data Supplement V.zip!/Data supplement 6-mRNAs targets of tsRNAs/tsRNA-Glu-TTC-010/Diagrams/tRF-Glu-TTC-010_vs_ENST00000321407(GCC1).pdf]
